# Genome Sequences of Three Strains of the *Pseudomonas aeruginosa* PA7 Clade

**DOI:** 10.1128/genomeA.01366-15

**Published:** 2015-11-19

**Authors:** Amine M. Boukerb, Romain Marti, Benoit Cournoyer

**Affiliations:** a Research Group on Bacterial Opportunistic Pathogens and Environment, UMR5557 Microbial Ecology, CNRS, VetAgro Sup and Université Lyon 1, Villeurbanne, France; b Biological Resource Centre–Environmental Microbiology Lyon (BRC-EML), UMR5557 Microbial Ecology, CNRS, VetAgro Sup and Université Lyon 1, Villeurbanne, France

## Abstract

Draft genome sequences of three *P. aeruginosa* strains from the PA7 clade are presented here. Their lengths are 6.36 (EML528), 6.44 (EML545), and 6.33 Mb (EML548). Comparisons with the PA7 genome showed 5,113 conserved coding sequences (CDSs), and significant numbers of strain-specific CDSs. Their analysis will improve our understanding of this highly divergent clade.

## GENOME ANNOUNCEMENT

*Pseudomonas aeruginosa* is a notorious opportunistic pathogen with intrinsic as well as acquired antibiotic resistances. It can provoke, among others, wound and burn infections, as well as acute and chronic respiratory infections, especially in cystic fibrosis patients (1). The *P. aeruginosa* pan-genome is known for its developed arsenal of virulence factors (2). PA7 is a clinical isolate from Argentina, which was found quite distinct from the other *P. aeruginosa* strains (2, 3). Gomila et al. (4) showed this genome to have diverged early from the other lineages through their phylogenomics analyses of the *Pseudomonas*. The genome sequences of the three PA7-related strains presented here will provide additional insights into the understanding of *P. aeruginosa* genomic organizations and its PA7 clade, especially regarding the evolutionary dynamics of genomic islands and other mobile elements playing a part in virulence and antibiotic resistances.

The strains were recovered from the EML-BRC collection of the French Network of Biological Resources Centers (FBRCMi; http://www.fbrcmi.fr). They were detected in the collection through *lecA*, *lecB*, and *ecfX* PCR screenings, sequencings, and DNA sequence comparisons (A. M. Boukerb et al., submitted for publication; [5]). The three selected strains had the following collection numbers: (a) EML528/CIP58.35 and (b) EML545/CIP59.45, initially reported by Kohler (6), and (c) EML548/CIP72.26, reported by Haynes (7). Genomic DNA was extracted from overnight bacterial broths according to Johnson et al. (8). Paired-end DNA sequences were generated by the HiSeq2000 Illumina system (Beckman Coulter, USA). The average insert size was about 260 bp (including 120 bp of adapters). Version 0.10.1 of FastQC (http://www.bioinformatics.babraham.ac.uk/projects/fastqc) was applied to check the quality of the generated 3,252,311, 3,261,408, and 3,181,166 reads, respectively. The paired-end reads were assembled *de novo* using the Mira genome assembler (9). Final assemblies contained 53, 62, and 120 contigs, with the largest ones being, respectively, 702,159 bp, 527,139 bp, and 307,235 bp. Total sequence spanning scaffolds were 6,365,411 bp, 6,442,109 bp, and 6,333,481 bp. Genome contigs were aligned against the PA7 genome sequence using the Mauve Contig Mover version 2.3.1 (http://asap.genetics.wisc.edu/software/mauve), and manual checks were done with ACT (Artemis Comparison Tool) at http://www.webact.org. The assembled genome sequences were processed through the Magnifying Genome platform using an automatic annotation pipeline (MaGe, http://www.genoscope.fr/agc/mage; [10]) and completed with manual checks and annotations. The assembled genomes contain, respectively, 6,409, 6,463, and 6,470 genomic objects, among which 6,281, 6,318, and 6,353 coding sequences could be detected. These genomes harbored four copies of the rRNA operons, 63 tRNAs, and 21 ncRNA genes. The GC contents were relatively close to that of the PA7 genome (~66.6%).

### Nucleotide sequence accession numbers.

The whole-genome shotgun projects have been deposited at DDBJ/EMBL/GenBank under the following accession numbers: LFXS00000000 for EML528, LGJE00000000 for EML545, and LFXR00000000 for EML548. The versions described in this paper are the first versions: LFXS01000000, LGJE01000000, and LFXR01000000, respectively.

## References

[1] Ratjen F, Döring G 2003 Cystic fibrosis. Lancet 361:681–689. doi:10.1016/S0140-6736(03)12567-6. 12606185

[2] Valot B, Guyeux C, Rolland JY, Mazouzi K, Bertrand X, Hocquet D 2015 What it takes to be a *Pseudomonas aeruginosa*? The core genome of the opportunistic pathogen updated. PLoS One 10:e0126468. doi:10.1371/journal.pone.0126468. 25961859 PMC4427113

[3] Roy PH, Tetu SG, Larouche A, Elbourne L, Tremblay S, Ren Q, Dodson R, Harkins D, Shay R, Watkins K, Mahamoud Y, Paulsen IT 2010 Complete genome sequence of the multiresistant taxonomic outlier *Pseudomonas aeruginosa* PA7. PLoS One 5:e8842. doi:10.1371/journal.pone.0008842. 20107499 PMC2809737

[4] Gomila M, Peña A, Mulet M, Lalucat J, García-Valdés E 2015 Phylogenomics and systematics in *Pseudomonas*. Front Microbiol 6:214. doi:10.3389/fmicb.2015.00214. 26074881 PMC4447124

[5] Lavenir R, Jocktane D, Laurent F, Nazaret S, Cournoyer B 2007 Improved reliability of *Pseudomonas aeruginosa* PCR detection by the use of the species-specific *ecfX* gene target. J Microbiol Methods 70:20–29. doi:10.1016/j.mimet.2007.03.008. 17490767

[6] Kohler W 1957 Zur Serologie der *Pseudomonas aeruginosa*. Z Immunforsch Exp Ther 114:282. 13478075

[7] Haynes WC 1951 *Pseudomonas aeruginosa*—its characterization and identification. J Gen Microbiol 5:939–950. doi:10.1099/00221287-5-5-939. 14908032

[8] Johnson WM, Tyler SD, Rozee KR 1994 Linkage analysis of geographic and clinical clusters in *Pseudomonas cepacia* infections by multilocus enzyme electrophoresis and ribotyping. J Clin Microbiol 32:924–930. 7517953 10.1128/jcm.32.4.924-930.1994 PMC263164

[9] Chevreux B, Pfisterer T, Drescher B, Driesel AJ, Muller WE, Wetter T, Suhai S 2004 Using the miraEST assembler for reliable and automated mRNA transcript assembly and SNP detection in sequenced ESTs. Genome Res 14:1147–1159. doi:10.1101/gr.1917404. 15140833 PMC419793

[10] Vallenet D, Belda E, Calteau A, Cruveiller S, Engelen S, Lajus A, Le Fevre F, Longin C, Mornico D, Roche D, Rouy Z, Salvignol G, Scarpelli C, Thil Smith AA, Weiman M, Medigue C 2013 MicroScope—an integrated microbial resource for the curation and comparative analysis of genomic and metabolic data. Nucleic Acids Res 41:D636–D647. doi:10.1093/nar/gks1194. 23193269 PMC3531135

